# Multidimensional regulatory mechanisms and translational potential of epigenetic networks in the rheumatoid arthritis disease course

**DOI:** 10.3389/fimmu.2026.1792863

**Published:** 2026-03-18

**Authors:** Yushan Zhang, Xianjun Xiao, Jing Guo, Xuantong Lei, Wen Xiong, Siying Wang, Yue He, Congjie Lei, Xiaoshen Hu

**Affiliations:** 1 College of Health and Rehabilitation, Chengdu University of Traditional Chinese Medicine, Chengdu, China; 2 The Affiliated Hospital of Chengdu University of Traditional Chinese Medicine, Chengdu, China; 3 Affiliated Sichuan Provincial Rehabilitation Hospital of Chengdu University of Traditional Chinese Medicine, Sichuan Provincial Ba-yi Rehabilitation Center, Chengdu, China; 4 Affiliated Zigong TCM Hospital of Chengdu University of TCM, Zigong, China

**Keywords:** autoimmune disease, DNA methylation, epigenetics, gut microbiota, histone modification, rheumatoid arthritis, RNA m6a

## Abstract

Rheumatoid arthritis (RA) is an autoimmune disease characterized by chronic synovitis that may progress to irreversible joint destruction and disability, thereby substantially impairing quality of life. RA results from complex interactions among genetic predisposition, environmental exposures, and immune dysregulation; however, current therapies are not curative, and many patients continue to experience pain, morning stiffness, and recurrent inflammation. In recent years, epigenetic mechanisms have emerged as key modulators of RA heterogeneity and disease persistence. Reversible regulatory layers—including non-coding RNAs, RNA modifications, DNA methylation, histone modifications, and microbiota–host interactions—provide a conceptual framework linking environmental cues to cell-type-specific inflammatory programs. This review summarizes recent advances in the epigenetic regulation of RA and outlines six interconnected dimensions. (1) miRNA-mediated post-transcriptional regulation: dysregulated miRNAs reshape inflammatory circuits and promote synovial activation through regulatory hubs. (2) RNA m^6^A modification: aberrant m^6^A remodeling alters immune metabolism and inflammatory gene expression, thereby reinforcing pathogenic responses. (3) DNA methylation: genome-wide profiling of synovium reveals differentially methylated loci that may activate disease-relevant pathways. (4) Histone modification and chromatin remodeling: altered activity of histone-modifying enzymes (e.g., HDACs) modulates inflammatory transcriptional programs and may contribute to epigenetic memory. (5) Hypoxia-driven metabolic–epigenetic crosstalk: hypoxia-inducible factors (HIFs) coordinate metabolic adaptation and inflammatory amplification; for example, HIF-1α supports the FLSs under hypoxic conditions. (6) Microbiome–epigenome interactions: gut microbial metabolites (e.g., butyrate) regulate immune homeostasis, partly by promoting follicular regulatory T cell (TFR) differentiation and restraining inflammation. Collectively, these findings indicate that epigenetic networks exert multilevel control over RA pathogenesis and highlight translational opportunities for targeted epigenetic interventions, including RNA methylation modulators, DNA methyltransferase inhibitors, and histone deacetylase–directed strategies.

## Introduction

Rheumatoid arthritis (RA) is an autoimmune disease that primarily affects the small joints, such as the proximal interphalangeal and metacarpophalangeal joints. Its main clinical manifestations include joint swelling, tenderness, and synovial inflammation. The 2010 RA diagnostic criteria (1) require that a patient must have at least one joint with definite swelling and a total score of 6 or higher from the following four domains:

- Number and location of affected joints (scoring range: 0-5).
- Serological abnormalities (scoring range: 0-3).
- Elevated acute-phase reactants (scoring range: 0-1).
- Duration of symptoms (scoring range: 0-2).

RA has a complex pathogenesis, and is recognized as a chronic autoimmune disorder driven by the interplay of genetic and environmental factors. This involves multiple elements, including genetic susceptibility (e.g., HLA-DR4), environmental triggers (such as smoking and infections), and epigenetic regulation (2). In recent years, epigenetics has drawn significant attention in RA pathogenesis research. RA affects nearly 1% of the global population (3), with a higher incidence in women (4). Current projections estimate that by 2050, the global RA patient population will reach 31.7 million. Epidemiological surveys in China show that there are approximately 5 million RA patients, with a prevalence rate of 0.42% (5). Data from the Chinese Registry of Rheumatoid Arthritis (CREDIT) indicate that around 9.5% of RA patients in China have comorbid major diseases, posing significant challenges for diagnosis and treatment.

Current RA treatment strategies focus on pain relief, inhibition of synovial cell proliferation, modulation of immune function, and protection of cartilage with the goal of controlling inflammation (6). The 2021 American College of Rheumatology guidelines for rheumatoid arthritis treatment (7) recommend the following: (1) conventional synthetic disease-modifying antirheumatic drugs (csDMARDs), such as methotrexate; (2) biological disease-modifying antirheumatic drugs (bDMARDs), for example, TNF-α inhibitors; (3) targeted synthetic disease-modifying antirheumatic drugs (tsDMARDs), like JAK inhibitors; (4) adjunctive glucocorticoid therapy (4).

Despite these available therapeutic approaches, some patients experience suboptimal efficacy, are susceptible to drug resistance, have high recurrence rates, and face an increased risk of complications. The pathological heterogeneity of RA suggests the presence of unresolved regulatory networks. Traditional genetic studies struggle to account for the reversibility of RA onset and significant individual differences, making epigenetic mechanisms a potential key to a breakthrough. Unlike conventional anti-inflammatory drugs, epigenetic interventions may directly reprogram the core drivers of pathological cells. Studies (8) have shown that ubiquitin-like containing PHD and RING finger domains 1 (UHRF1), a core epigenetic regulator, can suppress RA inflammation through negative feedback mechanisms and may serve as a therapeutic target. Roszkowski et al. (9) proposed that macrophages and monocytes driving inflammation in RA undergo polarization regulated by DNA methylation, miRNA, and histone modifications, thereby inducing inflammatory responses. They further suggested that targeting macrophages could halt inflammation and joint damage (10).

Epigenetics broadly refers to stable and potentially heritable changes in gene regulation and cellular phenotype that occur without alteration of the DNA sequence. In the context of RA, however, the relevant epigenetic alterations are predominantly somatic, tissue-specific, and context-dependent rather than transgenerational. Therefore, in this review, “heritability” mainly refers to mitotic stability (i.e., maintenance of cellular states during somatic cell division), which is particularly relevant to persistent pathogenic phenotypes in FLSs and immune cells.

Mechanistically, the core layers discussed here include DNA methylation, histone modifications/chromatin remodeling, non-coding RNA-mediated regulation, and RNA methylation (epitranscriptomic regulation). Hypoxia and microbiota-derived signals are discussed as upstream environmental and metabolic modulators that interact with and reshape these epigenetic programs.

First, mitotic stability refers to the faithful replication and transmission of epigenetic modifications during somatic cell division to daughter cells, thereby maintaining the functional phenotype of the same cell lineage (11). This is central to RA pathogenesis—abnormal DNA methylation, histone modifications, and other epigenetic alterations in synovial fibroblasts and immune cells drive “epigenetic clonal expansion” through localized cell proliferation, perpetuating and promoting the chronicity of inflammation.

**Figure 1:**
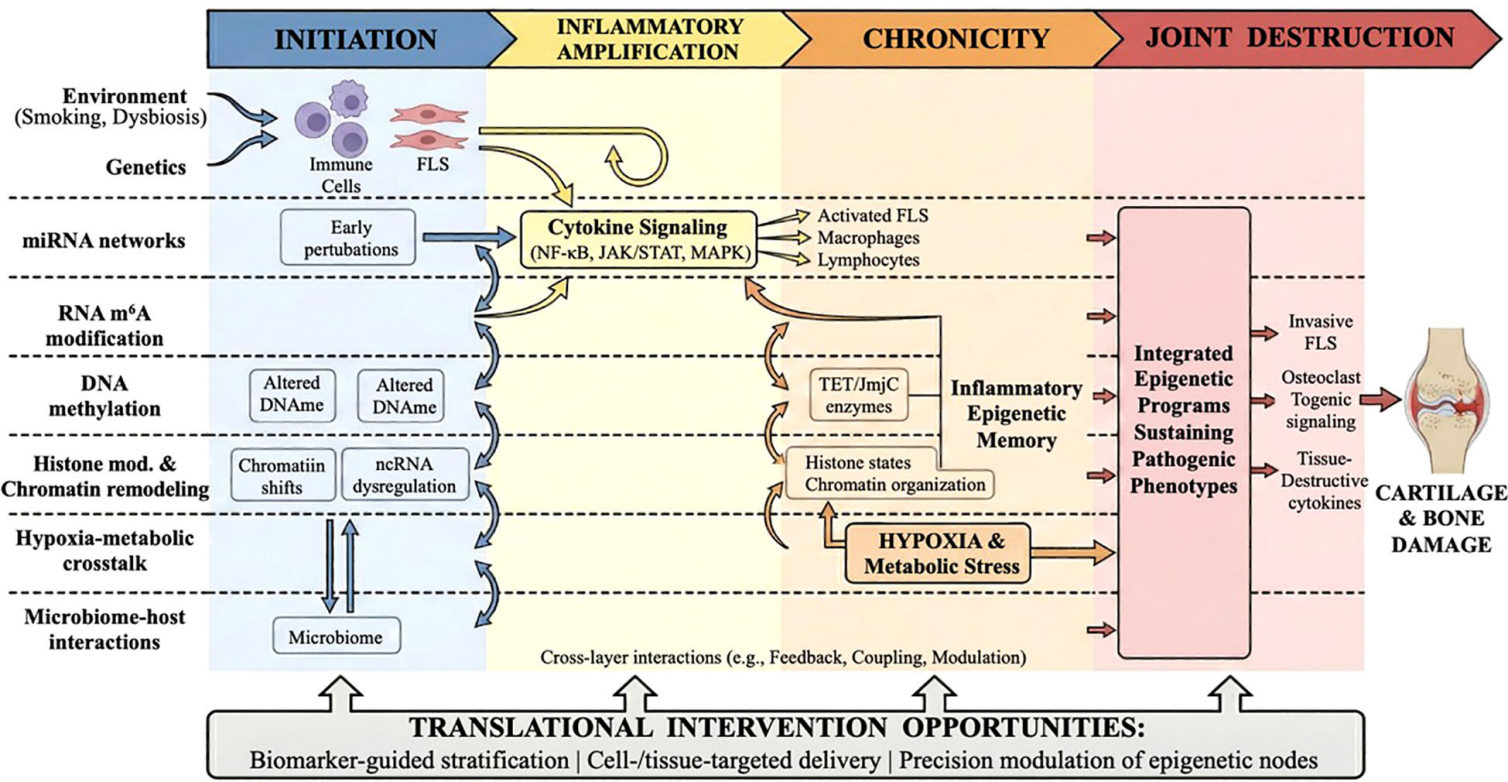
Systems-level model of cross-layer epigenetic regulation across the rheumatoid arthritis disease course.

The second mechanism is meiotic/transgenerational inheritance, where epigenetic modifications are transmitted to offspring via gametes. Except for a few imprinted genes and specific animal models exposed to particular environments, the vast majority of epigenetic abnormalities in RA patients are tissue-specific and occur at the somatic level, rather than being passed on to children through gametes. The RA epigenetic mechanisms discussed in this review are strictly confined to mitotic stability. (This clarification is provided because there is insufficient literature evidence to support otherwise.). Currently recognized mechanisms include microRNA, RNA methylation, DNA methylation, histone acetylation, hypoxia, and microbial factors. Among these, DNA methylation stands as one of the most extensively studied epigenetic modifications. See **Figure 1** for details.

This review intends to systematically analyze the dynamic imbalance of multi-level epigenetic regulatory networks during RA pathogenesis, thereby clarifying the underlying pathological mechanisms and therapeutic potential from the following six perspectives:

1. Non-coding RNA–transcription factor regulatory layer: To elucidate how miRNAs, together with other non-coding RNAs (e.g., lncRNAs/circRNAs), remodel synovial and immune-cell inflammatory programs by converging on core transcription factor pathways.
2. Post-transcriptional modification layer: To clarify the regulatory role of dynamic RNA m^6^A reprogramming in immune cell differentiation and synovial invasion.
3. Genomic epigenetic layer: To analyze the effect of aberrant DNA methylation patterns on immune cells and FLS invasion.
4. Chromatin remodeling layer: To reveal how histone modifications activate inflammatory responses by reshaping immune cell functions.
5. Metabolic stress layer: To examine how hypoxic microenvironments exacerbate the vicious cycle of synovial inflammation through epigenetic regulation.
6. Environmental interaction layer: To focus on the cross-species dialogues in which the gut microbiota and their metabolites influence the host’s immune homeostasis via epigenetic regulation.

In this review, we systematically dissect the multi-level epigenetic networks regulating RA pathogenesis through six interrelated dimensions: (1) miRNA-mediated transcriptional regulation, (2) RNA m^6^A reprogramming, (3) DNA methylation abnormalities, (4) histone modifications and chromatin remodeling, (5) hypoxia-driven metabolic-epigenetic cross-regulation, and (6) microbiome-epigenome interactions. Rather than functioning as six parallel mechanisms, the epigenetic layers discussed in this review are better understood as a dynamic, disease-course regulatory system that evolves across the major stages of RA (initiation → inflammatory amplification → chronicity → joint destruction). During the initiation phase, environmental exposures (e.g., smoking, mucosal/gut dysbiosis, and inflammatory triggers) interact with genetic susceptibility to establish early epigenetic perturbations, including altered DNA methylation patterns, chromatin accessibility shifts, and non-coding RNA dysregulation in immune and stromal cells. During inflammatory amplification, miRNA networks and RNA m6A remodeling reinforce cytokine signaling through pathway convergence (e.g., NF-κB, JAK/STAT, and MAPK modules), thereby promoting activation of FLS, macrophages, and pathogenic lymphocyte responses. As the disease progresses toward chronicity, persistent hypoxia and metabolic stress in the synovial microenvironment stabilize pathogenic transcriptional programs by influencing oxygen-sensitive epigenetic enzymes (e.g., TET and JmjC family dioxygenases), histone modification states, and chromatin organization, resulting in an “epigenetic memory” of inflammation. In the joint destruction stage, these integrated epigenetic programs sustain invasive FLS phenotypes, osteoclastogenic signaling, immune dysregulation, and tissue-destructive cytokine circuits, ultimately driving cartilage erosion and structural damage. This systems-level framework supports the central premise of this review: RA epigenetics should be interpreted as a spatiotemporally coupled network, not a collection of isolated molecular events. Together, this framework provides a mechanistic basis for interpreting cross-layer interactions across different stages of RA progression.

The schematic organizes six interconnected epigenetic layers—miRNA networks, RNA m6A modification, DNA methylation, histone/chromatin remodeling, hypoxia-driven metabolic–epigenetic crosstalk, and microbiome–host epigenetic interactions—across four disease stages (initiation, inflammatory amplification, chronicity, and joint destruction). Bidirectional connections represent cross-layer interactions that reinforce synovial inflammation, epigenetic memory, and tissue destruction. Potential translational entry points include biomarker-guided stratification, targeted delivery, and precision epigenetic modulation.

## Literature search strategy and review scope (narrative review)

This article is a narrative review with a structured literature retrieval process, rather than a systematic review or meta-analysis. Accordingly, the search and screening procedures were designed to improve topical coverage and transparency, but they do not fully meet PRISMA standards (e.g., fully reproducible search strings for all databases, formal risk-of-bias assessment, and a systematic evidence synthesis workflow).

Retrieved records were imported into EndNote 21 for deduplication, yielding 464 non-duplicate records (database retrieval status as of February 25, 2026). The first author conducted title/abstract screening to exclude clearly irrelevant publications, after which 223 articles were retained for full-text assessment. Full texts were then reviewed for relevance to rheumatoid arthritis epigenetic regulation, mechanistic contribution, and suitability for inclusion in a narrative synthesis.

Articles selected for full-text review were managed in EndNote using a custom group (e.g., “Full Text Assessed”), while full-text files were accessed via institutional subscriptions or local storage and were not uniformly attached within EndNote. Because the screening records were retrospectively reconstructed during revision, minor discrepancies in intermediate counts may exist. To avoid overstating methodological rigor, we present these counts as a transparent reconstruction of the review workflow rather than a PRISMA-compliant systematic screening record.

The final reference list includes original research articles, reviews, and meta-analyses. Reviews/meta-analyses were used primarily for background framing and conceptual synthesis, whereas mechanistic interpretations in the main text were based predominantly on original research studies. Inclusion was guided by mechanistic relevance to RA epigenetic regulation, representativeness of key pathways/cell types, and evidentiary value, rather than journal quartile alone. We acknowledge that this narrative approach may introduce selection bias and that no formal risk-of-bias scoring was performed.

## Non-coding RNA–transcription factor regulatory layer: miRNA-centered network dysregulation in RA

MicroRNAs (miRNAs) are a class of non-coding, single-stranded RNA molecules. These small structures, composed of approximately 18–22 nucleotides, are key players in the transcription process and gene expression regulation. Normally, RNA polymerase II transcribes miRNAs from the genome to produce precursor miRNAs. After undergoing two successive rounds of processing and cleavage, these precursors mature into functional miRNAs, which are about 21 nucleotides in length (12). They typically bind to complementary sequences in target mRNAs (most commonly within the 3′ untranslated region), thereby regulating mRNA stability and/or translation efficiency (13, 14). A single miRNA can have one or more mRNA targets. By specifically targeting and inhibiting mRNAs related to the onset of RA pathogenesis, especially those encoding pro-inflammatory factors, miRNAs impact multiple biochemical processes in the human body, such as cell differentiation, proliferation, homeostasis, and immune responses (15). When miRNA expression becomes dysregulated, it can trigger inflammatory signaling pathways. This serves as an early trigger for the abnormal activation of FLS and oint destruction. Existing research reveals that miRNA regulation in RA exhibits characteristics of multi-target, multi-pathway, and cell-type specificity. See **Table 1** for details. To better reflect the mechanistic scope of this layer, it should be emphasized that RA-related non-coding RNA regulation is not limited to miRNAs alone. In this review, miRNAs are discussed as the central component of this layer because they currently have the most extensive functional evidence in RA. However, other non-coding RNAs—particularly long non-coding RNAs (lncRNAs) and circular RNAs (circRNAs)—also participate in inflammatory regulation by acting as competing endogenous RNAs (ceRNAs), scaffolds for chromatin/transcriptional regulators, or modulators of signaling pathway output. These ncRNA species converge on core transcription factor programs (e.g., NF-κB, STATs, and AP-1), thereby shaping cell-type-specific inflammatory phenotypes in FLS, macrophages, and T cells.

**Table 1:** miRNA and RA.

Author	miRNA	Cell or tissue	Expression changes	Function	Promotes/inhibits RA
Bae et al.	miRNA-146	Synovial tissue	Up-regulation	Associated with RA inflammatory processes	Promotes
Zhu et al.	miRNA-99b-5p	T cells	Up-regulation	Inhibit T cell apoptosis, stimulate their proliferation and activation, and promote proinflammatory factor expression	Promotes
O’Connell et al.	miRNA-155	Th17 cells	Up-regulation	Promote autoimmune inflammatory responses	Promotes
Kurowska-Stolarska et al.	miRNA-155	CD14+ cells	Up-regulation	Induces production of cytokines and chemokines closely associated with RA	Promotes
Renman et al.	miRNA-26b	FLS	Down-regulation	Increases TNF-α, IL-1β, and IL-6, inducing inflammatory response	Promotes
Pauley et al.	miRNA-146a	FLS	—	Inhibits the destruction of bone and cartilage.	Inhibits
Llop et al.	miRNA	Immune cells	—	Increases the risk of cardiovascular diseases.	No obvious tendency

At the target gene network level, miR-155—the most extensively studied pro-inflammatory miRNA in RA—directly targets the mRNAs of SOCS1 and SHIP1. Studies demonstrate that in peripheral blood mononuclear cells from RA patients, miRNA-155 targets and suppresses SOCS1, thereby upregulating inflammatory mediators such as TNF-α (16). In RA FLS, miRNA-155-5p similarly negatively regulates SOCS1 expression, promoting cell proliferation and invasion (17). This releases its inhibition on the JAK-STAT and PI3K-Akt pathways. Conversely, miR-146a exerts anti-inflammatory effects by negatively regulating the TLR/NF-κB signaling pathway through targeting IRAK1 and TRAF6. These miRNAs form complex miRNA-mRNA regulatory networks with their target genes, rather than simple one-to-one relationships.

At the convergence point of signaling pathways, multiple RA-associated miRNAs jointly regulate three core inflammatory pathways: miR-155, miR-21, and miR-181a can activate the NF-κB pathway through different targets; miR-155 and miR-146a influence the JAK-STAT pathway by regulating SOCS1 and IRAK1; while miR-132 and miR-223 participate in MAPK pathway regulation. This “multiple miRNAs-single pathway” convergence model enables sustained amplification of inflammatory signals within the RA synovial microenvironment.

At the cell type-specific level, the same miRNA exerts distinctly different functions in various immune cells and stromal cells. For example, miR-155 promotes Th17 differentiation in T cells, drives M1 polarization in macrophages, and enhances proliferation and invasion in FLS (17, 18). miR-146a exhibits protective effects through elevated expression in T cells of early-stage RA patients, but its downregulation in advanced FLS leads to loss of anti-inflammatory regulation. This cell-type-dependent functional divergence elucidates the complexity of miRNA regulation in RA and the challenges for therapeutic targeting.

**Figure 2:**
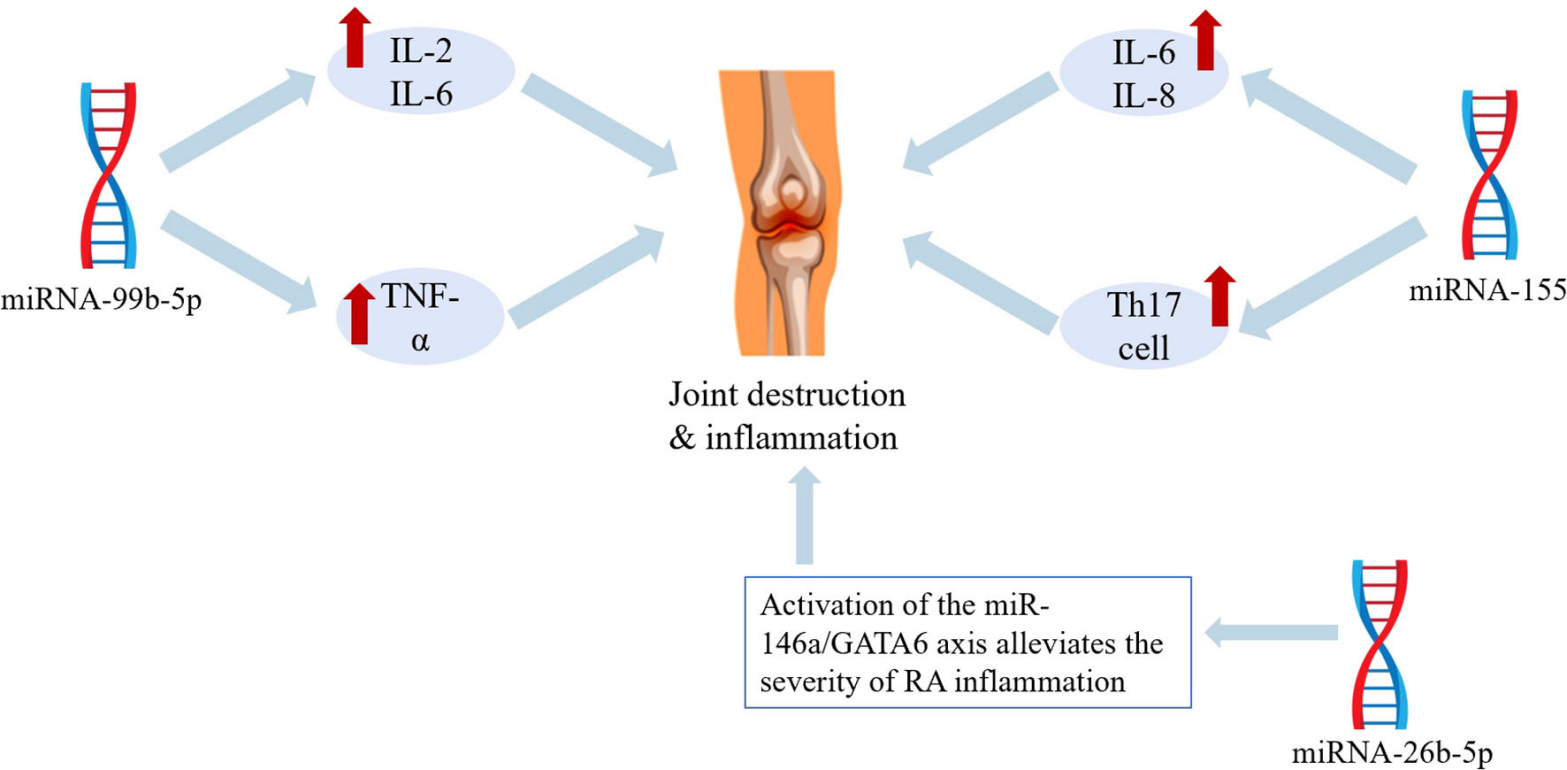
Schematic diagram of miRNA pathogenic mechanisms.

In studies on how miRNAs contribute to RA inflammation, Bae et al. (19) made an important discovery. Through their experiments, they found that the levels of miRNA-146 in RA patients were significantly higher than those in healthy individuals. Other research has also pointed out that miRNA-146 levels are elevated in the synovial tissue of RA patients compared to non-RA controls, indicating its possible involvement in RA pro-inflammatory processes. Zhu et al. (20) found that miRNA-99b-5p expression was upregulated in RA patients, particularly in T cells. The levels of this miRNA in T cells of RA patients were far higher than those in healthy individuals. This miRNA can reduce cell death rates, stimulate cell division and growth, and increase the production of pro-inflammatory factors like IL-2, IL-6, TNF-α, and IFN-γ. These actions collectively drive the synovial inflammatory cascade and contribute to the development of RA. O’Connell et al. (18) discovered that miRNA-155 promotes autoimmune inflammatory responses and is involved in the development of Th17 cells during tissue inflammation, which in turn promotes T cell-dependent tissue inflammation. Consequently, miRNA-155 may be a potential therapeutic target for autoimmune diseases. Kurowska-Stolarska et al. (21) demonstrated that miRNA-155 is highly expressed in CD14+ cells from RA synovium. It activates cytokines and chemokines closely associated with RA, such as TNF-α, IL-6, and IL-8, causing synovial inflammation (22). In terms of reducing RA inflammation, Pauley et al. (23) proposed that miRNA-146a could serve as a therapeutic target. Activating the miR-146a/GATA6 axis can decrease the invasive ability of FLS, thus partially preventing bone and cartilage damage. Renman’s group (24) found that the expression of miRNA-26b-5p was lower in RA patients and their first-degree relatives than in healthy controls. miRNA-26b is related to RA inflammatory responses, and its suppression in RA FLS leads to higher levels of TNF-α, IL-1β, and IL-6 (25). Regarding RA-related complications, Llop et al. (13) suggested that individuals with RA are more likely to develop carotid plaque (cPP) because of the presence of miRNAs and chronic inflammation. This condition causes problems with the inner lining of blood vessels, the accumulation of immune cells in plaques, and plaque instability, increasing the risk of cardiovascular diseases like atherosclerosis (14). See **Table 1**, **Figure 2** for details.

Beyond miRNAs, lncRNAs and circRNAs contribute to RA pathogenesis by rewiring ncRNA–mRNA–transcription factor networks. A representative mechanism is ceRNA regulation, in which lncRNAs/circRNAs sequester specific miRNAs and thereby release repression of downstream targets. For example, lncRNA LINC-PINT has been reported to increase SOCS1 expression by sponging miR-155-5p in TNF-α-induced RA synovial fibroblasts, suppressing ERK signaling and attenuating inflammatory activation. This illustrates that ncRNA regulation in RA is not a linear “single miRNA–single target” process, but rather a layered regulatory circuit involving upstream ncRNA sponges, downstream target genes, and convergent inflammatory pathways.

In addition, some lncRNAs may influence inflammatory transcription more directly by interacting with chromatin-associated proteins or transcriptional complexes, although RA-specific causal evidence remains heterogeneous across studies. Therefore, the ncRNA layer in RA should be understood as a broader regulatory network in which miRNAs, lncRNAs, and circRNAs collectively modulate transcription factor activity and inflammatory signaling outputs in a context-dependent manner.

## Post-transcriptional modification layer: RNA m^6^A methylation reprogramming drives synovial invasion in rheumatoid arthritis (RA)

RNA methylation is carried out by methyltransferases. These enzymes selectively attach methyl groups to RNA molecules after transcription, thereby helping to regulate gene expression. Among all known chemical modifications of RNA molecules, methylation is the most common form (26, 27). RNA methylation has a significant influence on the differentiation of immune cells and the synovial invasive phenotype in RA. The abnormal expression of methyltransferases across spatial and temporal contexts creates the foundation for epigenetic memory. This may contribute to irreversible pathological damage during the chronic course of RA. See **Table 2** for additional details.

**Table 2:** RNA methylation and RA.

Author	miRNA	Cell or tissue	Expression changes	Role	Promote/inhibit RA
Miao et al.	METTL3	FLS	Up-regulation	Through the METTL3-m^6^A-YTHDC2-AMIGO2 axis, it promotes mRNA degradation and enhances the invasive and proliferative capacity of FLS.	Promote
Tong et al.	METTL3	Macrophage	Up-regulation	m^6^A modification enhances IRAK-M translation, activates an alternative NF-κB pathway, and produces inhibitory molecules.	Inhibit
Xu et al.	METTL14、METTL3	Macrophage、FLSs	Down-regulation	Promotes macrophage polarization, generates pro-inflammatory effects, activates FLSs, and facilitates their proliferation.	Promote
Xu et al.	METTL3-METTL14 Complex	Macrophage	Up-regulation	Regulates the inflammatory response in some macrophages.	Inhibit
Li et al.	FTO、ADAMTS15	FLS	Up-regulation	Promotes bone destruction and cartilage erosion, triggering inflammatory responses.	Inhibit
Kim et al.	FTO	NK cells	Down-regulation	Regulating m^6^A modification of SOCS family members affects the JAK-STAT pathway and enhances NK cell activity.	Promote

**Figure 3:**
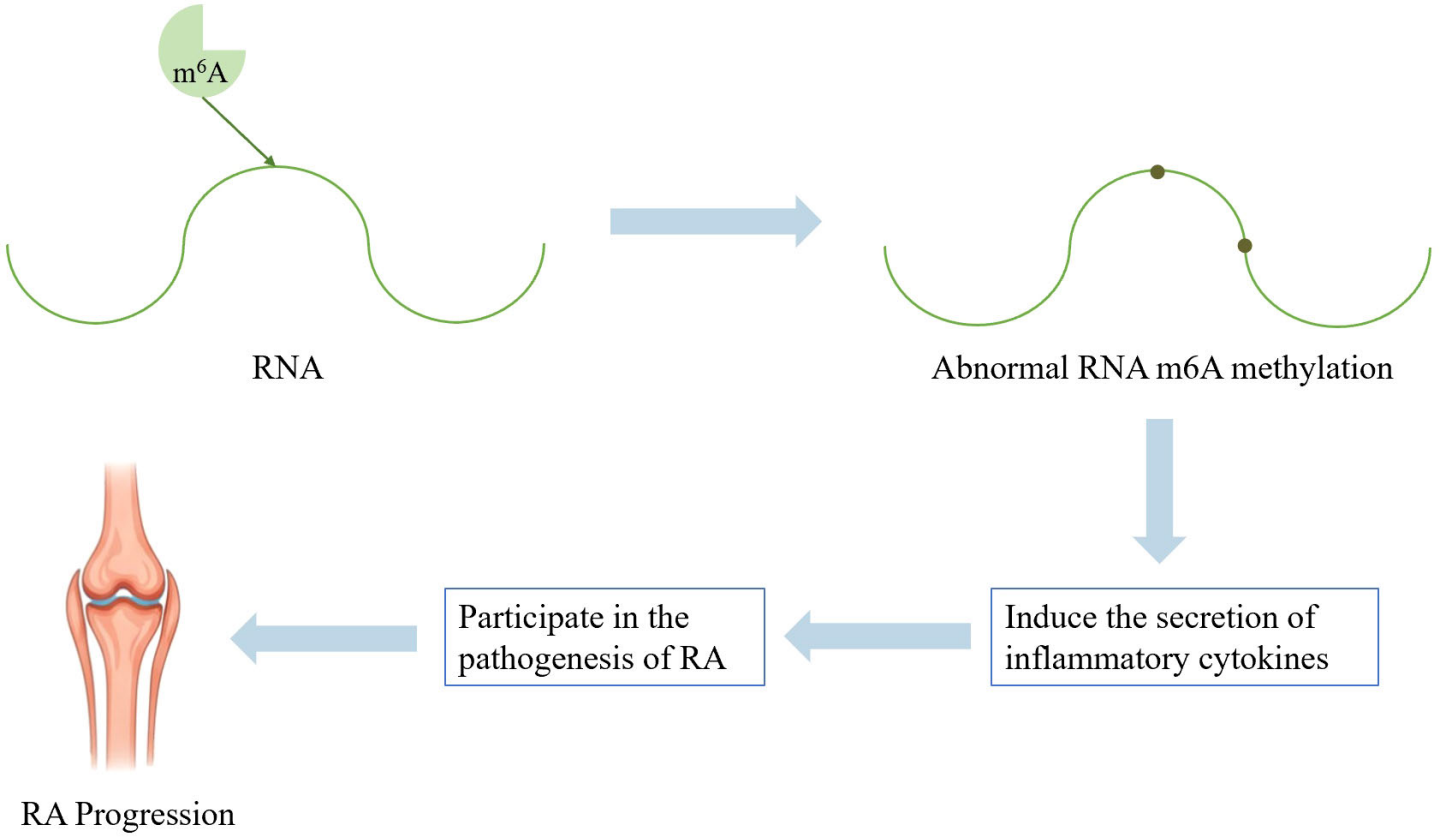
Schematic diagram of RNA methylation pathogenesis mechanism.

N6-adenylate methylation (m^6^A) stands out as the most thoroughly investigated mRNA modification and serves as the primary form of mRNA methylation in mammals. RNA m^6^A modification is reversible. It can influence the post-transcriptional fate of modified RNA molecules and regulate many essential biological processes, including aberrant immune activation (28, 29). Aberrant m^6^A methylation contributes to the occurrence of RA by regulating synovial fibroblasts and cell-cell interactions, as well as by promoting the secretion of inflammatory cytokines. It interacts with key immune cells, including macrophages, dendritic cells, and lymphocytes, which in turn initiates joint inflammatory responses (28, 30). See **Figure 3** for further details.

RNA m^6^A methylation regulation in RA exhibits cell type-dependent and dynamic equilibrium characteristics. m^6^A modification levels are determined by the coordinated action of three classes of proteins: writers such as the METTL3/METTL14 complex catalyze m^6^A formation; erasers such as FTO and ALKBH5 remove methyl groups; and readers such as the YTHDF family proteins recognize m^6^A modifications and influence mRNA stability, splicing, or translation (31–35). Differences in the expression levels and activities of these three types determine the final effects of m^6^A modification in different cell types.

In synovial FLSs, high METTL3 expression promotes mRNA degradation through the METTL3-m^6^A-YTHDC2-AMIGO2 axis, enhancing FLS invasion and proliferation capabilities (35–37). In contrast, the effects of METTL3 in macrophages may be entirely different: studies indicate that METTL3 enhances the translation of IRAK-M (a negative regulatory molecule) via m^6^A modification, activating an alternative NF-κB pathway. This leads to the production of inhibitory molecules (such as SOCS1, SHIP1, A20, and IκBα), thereby suppressing inflammation (38, 39). Xu et al. (40) proposed the following insights: downregulation of METTL14 in peripheral blood mononuclear cells (PBMCs) drives inflammatory cytokine secretion; in macrophages, METTL3 mediates the polarization of synovial M1 macrophages; and in FLS, METTL3 and METTL14 may jointly participate in activating FLS and promoting their proliferation. Xu et al. (41) demonstrated that levels of the METTL3-METTL14 complex—the most critical subunits within the m^6^A methyltransferase complex (MTCs)—are upregulated in RA patients. This upregulation regulates the nuclear translocation of phosphorylated nuclear factor κB (NF-κB), thereby suppressing inflammatory responses in certain macrophages. Therefore, RA may be treatable by activating the METTL3/NF-κB signaling pathway. This difference arises from distinct mRNA pools targeted by METTL3 in different cells and variations in reader/eraser expression profiles.

Furthermore, the role of the eraser FTO in RA exhibits cell-type dependency: Li et al. (42) demonstrated that the fat mass and obesity-associated protein (FTO) gene is overexpressed in FLS and synovium from RA patients. High FTO expression mediates m^6^A modification via IGF2BP1, reducing ADAMTS15 stability and expression levels to promote inflammation in RA FLS. Conversely, in T cells, FTO may influence the JAK-STAT pathway by regulating m^6^A modification of SOCS family members, thereby enhancing NK cell activity (43). These findings suggest that m^6^A regulation constitutes a dynamic, cell-specific system rather than a simple pro- or anti-inflammatory switch. Future studies should integrate single-cell technologies to decipher the precise expression profiles of writer/eraser/reader enzymes across distinct cell subpopulations, thereby elucidating the complete m^6^A regulatory network in RA.

Current m^6^A modification studies in RA primarily rely on FLS cell lines and CIA mouse models. The role of METTL3 in macrophages remains controversial, and single-cell resolution data from human primary cells are lacking. Research on FTO in RA is still in its infancy, and its functional heterogeneity across different immune cell subpopulations requires systematic investigation.

## Epigenomic layer: abnormal DNA methylation shapes the pro-inflammatory epigenome of FLS

DNA methylation stands as the most thoroughly investigated epigenetic modification. It is catalyzed by DNA methyltransferases (DNMTs), which attach methyl groups to cytosine-phosphate-guanosine (CpG) dinucleotides. CpGs grouped in gene promoter regions are key regulators of gene transcription (44). By changing the chromatin structure, they can ultimately silence genes. This process prevents monocytes and macrophages from generating pro-inflammatory mediators, such as the S100 calcium-binding protein family, cytokines, chemokines, and metalloproteinases. These pro-inflammatory mediators can seep into the synovial membrane, leading to tissue damage and inducing pain and discomfort in patients (45). There is a direct association between this epigenetic imbalance and the heightened invasiveness of FLS as well as immune dysfunctions in autoreactive T cells. DNA methylation signatures may be found in the early stage of disease, opening up opportunities for prompt intervention and symptom alleviation. See **Table 3** for details.

**Table 3:** DNA methylation and RA.

Author	DNA methylation	Cell or tissue	Expression changes	Role	Promote/inhibit RA
Martín-Núlez et al.	DNA-specific CpG site methylation levels	—	Up-regulation	Biomarkers for diagnosing RA	No clear tendency
Sun et al.	MeCP2、PTCH1 genes	Synovial tissue	MeCP2 Up-regulation、PTCH1 Down-regulation	Associated with RA pathogenesis	No clear tendency
Fang et al.	CCDC88C gene	FLS	Down-regulation	Induce or influence RA development	Promote
Chen et al.	PI3K-Akt Signaling pathway	FLS、Synovial cells and Osteoclast	Up-regulation	FLS metabolism and promotion of synovial cell and osteoclast proliferation	Promote
Webster et al.	LRPAP1 gene	—	—	Potential therapeutic target for RA, with a pathway linked to established RA treatment biomarkers	Inhibit
Huang et al.	FOXP3 promoter region	Treg cells	Up-regulation	Silencing of FOXP3 protein expression impairs the immunosuppressive function of Tregs.	Promote
Ling et al.	GPX4 promoter	FLS and Synovial tissue	Up-regulation	Reduces GPX4 levels, inhibits ferroptosis, alleviates inflammation, and slows RA progression	Inhibit

The regulatory function of DNA methylation in RA exhibits strict regional specificity: high methylation in promoter regions correlates with transcriptional silencing, while methylation in genomic regions predominantly enriches within active transcribed genes, participating in transcription elongation and splicing regulation. This section will separately discuss research progress and existing gaps regarding these two methylation patterns in RA.

**Figure 4:**
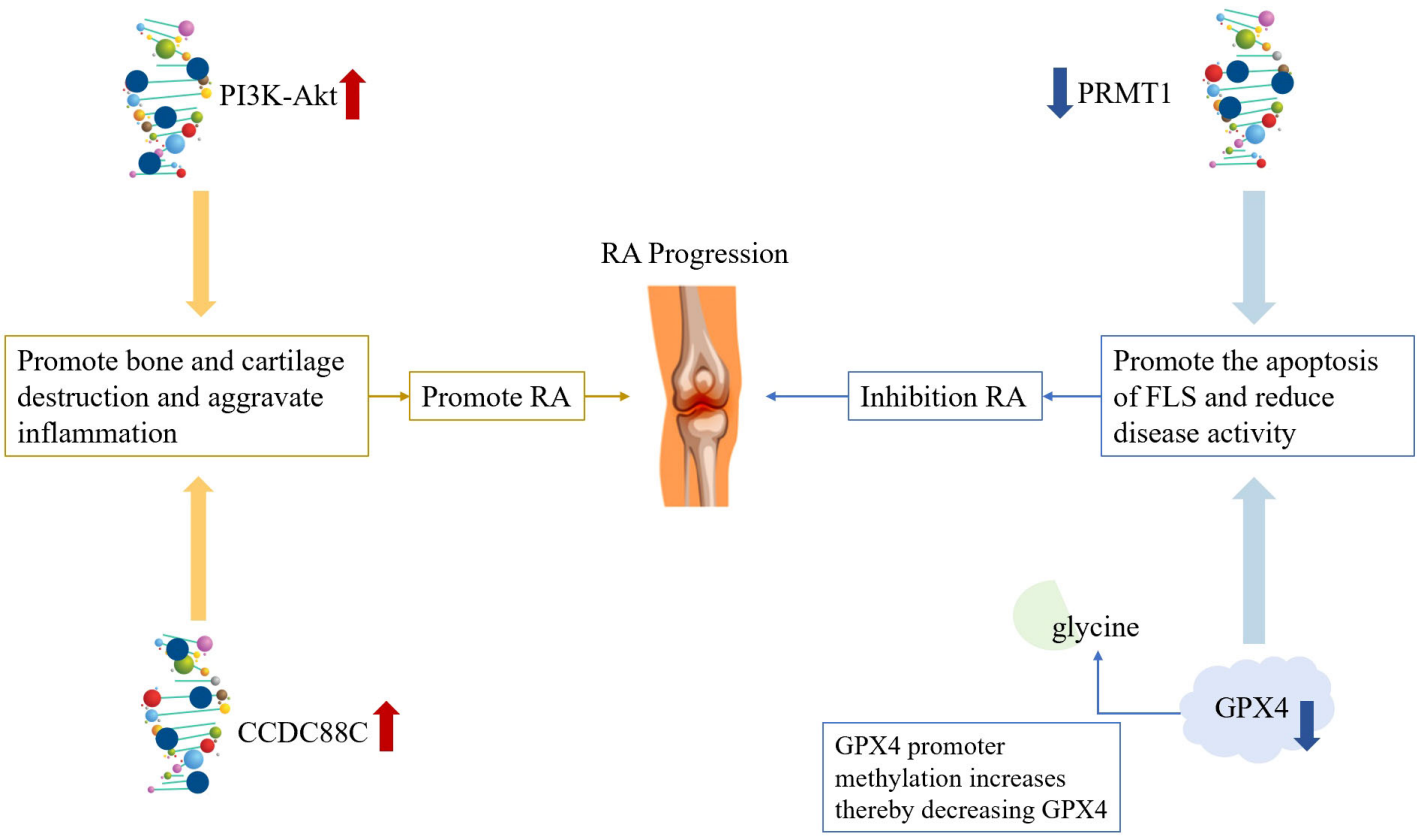
Schematic diagram of the pathogenic mechanism of DNA methylation.

At the promoter level, characteristic methylation abnormalities are observed in RA FLS and immune cells, such as those reported by the Martín-Núñez team, who (46) performed genome-wide DNA methylation studies on severe RA samples. Their results revealed that these samples presented with higher average DAS28-ESR scores, a larger quantity of Collinsella, a more frequent occurrence of erosion, and elevated anti-citrullinated peptide antibodies (ACPA) levels. They proposed that CpGs located in differentially methylated regions, along with other CpGs, could serve as potential biomarkers. Fang et al. (47) identified four genes—FARLA, CCDC88C, BCL11B, and APOL6—that showed hypomethylation and down-regulation in RA compared to osteoarthritis (OA) samples. Among these genes, CCDC88C (coiled-coil domain containing 88C), which plays a role in regulating the Wnt signaling pathway, may trigger or impact RA pathogenesis via this pathway (48). Sun and his team (49) reported that in the synovial tissue of AA rats, MeCP2 expression was up-regulated while PTCH1 expression was down-regulated. Following treatment with the methylation inhibitor 5-azadc, the expression of both MeCP2 and PTCH1 declined. Furthermore, MeCP2 regulates the PTCH1 gene by binding to methylated CpG sites within the PTCH1 promoter region. The discovery that MeCP2 regulates PTCH1 highlights its pivotal role in the pathogenesis of Rett syndrome. In terms of promoting RA inflammation, Chen et al. (50) proposed that DNA methylation activates the PI3K-Akt signaling pathway, which affects FLS metabolism and promotes the proliferation of synovial cells and osteoclasts, thereby worsening patient symptoms. As for suppressing RA inflammation, the Webster team (51) experimentally identified LRPAP1 as the gene most closely related to RA. The protein produced by this gene is associated with low-density lipoprotein receptor activity, and its methylation is a potential therapeutic target for RA. They also discovered that the PRKCZ gene shares related pathways with LRPAP1 and has been established as a biomarker for RA treatment (52). The methylation status of the FOXP3 gene promoter directly determines the functional integrity of regulatory T cells (Tregs). Studies indicate that the FOXP3 promoter region in Tregs from peripheral blood and synovial tissue of RA patients exhibits relatively high methylation, leading to silencing of FOXP3 protein expression and impaired Treg immunosuppressive function (53). This epigenetic silencing can be reversed by DNA methyltransferase inhibitors (e.g., decitabine) or by immunomodulatory interventions (e.g., rapamycin), potentially restoring Treg function and stability (54). Notably, FOXP3 promoter methylation exhibits synergistic regulation with histone acetylation modifications (detailed in Section 4), illustrating cross-level interactions among epigenetic modifications. Ling et al. (55) observed that ferroptosis, a non-apoptotic cell death pathway, is reduced in RA FLS and synovium, which exacerbates inflammation and accelerates RA progression. Research (56) has demonstrated that glutathione peroxidase 4 (GPX4) can inhibit the ferroptosis process. Glycine promotes increased methylation of the GPX4 promoter, while overexpression of the methylating enzymes DNMT1/3A/3B reduces GPX4, thereby increasing ferroptosis and improving RA progression. It should be noted that the aforementioned studies on promoter methylation exhibit significant disparities in the level of evidence. The differentially methylated regions identified through genome-wide association studies primarily provide correlational evidence, and direct validation of their causal relationship with disease remains to be conducted. Functional studies on genes such as CCDC88C and MeCP2 are mainly based on animal models or *in vitro* cell experiments; their actual role in RA patients requires further validation using human primary cells. See **Figure 4** for details.

In contrast, research on genomic methylation in RA remains in its infancy. Unlike the transcriptional suppression effect of promoter methylation, genomic methylation primarily enriches in the coding regions of highly expressed genes, functioning to regulate transcription elongation efficiency and alternative splicing (57). The underlying mechanism involves active gene promoter regions actively avoiding DNA methylation due to their enrichment in H3K4me3 modifications. whereas genomic regions recruit H3K36me3 modifications, whereas genomic regions recruit H3K36me3-modified histones during RNA polymerase II passage in transcription elongation. These histones are specifically bound by the PWWP domain of DNMT3B, inducing programmed hypermethylation in genomic regions (57–59). This synergistic modification pattern ensures the “segregated coexistence” of low methylation at promoters and high methylation in gene bodies. Its functional roles include: (1) Suppressing latent promoter activity—gene bodies harbor potential endogenous promoter sequences; moderate methylation prevents abnormal transcription initiation, ensuring fidelity of full-length transcripts; (2) Regulation of transcription elongation kinetics—nucleosome deposition and methylation at genomic boundaries induce transient pauses in RNA polymerase II, a process coupled with spliceosome recruitment that influences exon fusion patterns (58, 60). Within the RA research field, gaps in genomic methylation studies are particularly pronounced. Current methylation chip studies of RA synovial tissue almost exclusively focus on promoter regions, with severely inadequate annotation of differentially methylated genomic regions. Considering the coexistence of global hypomethylation and localized hypermethylation in FLS, future research should systematically analyze the genomic methylation status of effector genes such as FOXP3, MeCP2, and PTCH1, along with their associations with abnormal transcription elongation and mRNA splicing variants. Furthermore, DNMT inhibitors like decitabine used in RA treatment exploration must be approached with caution due to their “double-edged sword effect”—while reversing promoter hypermethylation, they may cause tumor suppressor gene expression to initially rise before rapidly declining, potentially leading to drug resistance (61).

## Chromatin remodeling layer: dysregulation of the histone modifier enzyme network and pathological remodeling in RA

Histone modifications are mainly regulated by lysine acetyltransferases, histone deacetylases, lysine methyltransferases, and lysine demethylases (62). When it comes to research on RA, most studies have centered on histone acetylation, which is mediated by histone acetyltransferases (HATs). The retinoic acid-related orphan receptor gamma t (RORγt) gene may take part in the differentiation of Th17 cells and can stimulate these cells to produce pro-inflammatory mediators. This, subsequently, worsens the infiltration of inflammatory cells in the choroid plexus and harms the joints (22, 63–65). Regulatory T cells (Tregs) can inhibit the proliferation and activity of Th cells through direct contact and the secretion of anti-inflammatory cytokines. The key transcription factor that controls Tregs is Foxp3, which maintains the suppressive activity of mature peripheral Tregs (66). A reduction in the activity of Foxp3 can result in Tregs changing into Th17 cells (67). Tip60, an HIV tat-interacting protein, functions as a histone acetyltransferase. It not only has an impact on Treg differentiation but also influences the co-regulation of Treg and Th17 cells (68). Tip60 interacts with Foxp3 to suppress RORγt expression (69). Imbalances in the modification enzyme network mediated by HDACs not only contribute to the persistence of chronic inflammatory microenvironments in RA synovial tissue but also worsen therapeutic resistance through epigenetic memory. See **Table 4** for details.

**Table 4:** Histone modification and RA.

Author	Histone modification	Cell or tissue	Expression changes	Role	Promote/inhibit RA
Su et al.	Tip60、Foxp3	Treg cells、CD4+T cells	Down-regulation	Inhibits Foxp3 expression and Treg differentiation, thereby inducing inflammation	Promote
Krošel et al.	CBP	—	Up-regulation	Induce anti-inflammatory effects and specifically downregulate genes associated with RA disease activity and prognosis prediction.	Inhibit
Okamoto et al.	MLL1	RASFs	Up-regulation	Promote inflammatory responses.	Promote
Lin et al.	HATs	Th17 cells	Down-regulation	Inhibits RORγt expression in Th17 cells, thereby suppressing inflammation	Inhibit
Liu et al.	PRMT1 gene	FLS	Up-regulation	Inhibits FLS proliferation, invasion, and migration in RA, promotes FLS apoptosis, thereby alleviating the inflammatory response	Inhibit
Park et al.	HDAC6	FLS	Up-regulation	Promote bone and cartilage damage as well as FLS-mediated inflammatory responses.	Promote
Li et al.	SPRC	FLS	Down-regulation	Inhibits HDAC6 gene expression and its induced inflammatory genes	Inhibit
Loh et al.	H3K27ac	FLS	Up-regulation	Through sustained chromatin activation, FSGs evade transcriptional repression, forming epigenetic memory that drives chronic synovitis.	Promote
Hammarker et al.	IL-6、IL-6R and JAK1 gene	FLS	Up-regulation	Joint-specific chromatin states may influence the intensity of FLS responses to cytokines by regulating the accessibility of the JAK-STAT pathway.	Promote
Ai et al.	BRD4	FLS	Up-regulation	Drive the explosive expression of core pro-inflammatory genes such as IL-6 and IL-1β	Promote
Ge et al.	Hi-C/CHiC、Enhancer-Promoter Interaction Network	FLS	Positively correlated with changes in the expression of neighboring genes	Establishing a novel “accessibility platform” through reconstruction of the enhancer-promoter interaction network—upregulated gene regions enrich AP-1 binding sites, while downregulated gene regions enrich BACH2 and homobox/forkhead box family transcription factor binding sites.	Promote

Concerning the promotion of RA inflammation, Su et al. (69) demonstrated that Treg cell production was reduced in CD4^+^ T cells collected from RA patients, accompanied by decreased expression of Tip60 and Foxp3. Restoring Tip60 expression reduced Th17 cell numbers and inflammation. Krošel et al. (70) proposed that silencing calmodulin-binding protein (CBP) can generate anti-inflammatory effects and specifically downregulates genes associated with RA disease activity and prognostic prediction (71). Silencing p300 gene expression can trigger both pro-inflammatory and anti-inflammatory responses, as well as responses that contribute to tissue damage. Okamoto et al. (72) demonstrated abnormal mRNA expression of histone lysine methyltransferases (HKMTs) in RA SFs. For example, myeloid/lymphoid or mixed-lineage leukemia 1 (MLL1) (73), is highly expressed in RASFs. Deletion of this gene reduces the levels of histone H3 lysine 4 trimethylation (H3K4me3) in the promoters of cytokines and chemokines within RASFs, thereby suppressing the expression of cytokine and chemokine, including IL-6, IL-15, CCL2, and CCL5. Thus, abnormal expression of this gene accelerates the synovial inflammatory cascade. Lin et al. (74) found that urushiol, a non-specific inhibitor of the histone acetyltransferase (HAT) family, can suppress RORγt expression in Th17 cells, thereby inhibiting inflammation. Liu’s work (75) proposed that protein arginine methyltransferase 1 (PRMT1) catalyzes the methylation of multiple substrates, including histones and non-histones, thereby facilitating arginine methylation. They found that PRMT1 protein levels were higher in RA synovial tissue than in control synovial tissue. PRMT1 knockout out of the PRMT1 gene significantly curbed FLS proliferation, invasion, and migration in RA, promoted FLS apoptosis, and reduced disease activity. Park et al. (76) found that inhibiting histone deacetylase 6 (HDAC6) in FLS can reduce cytokine production, which in turn weakens inflammatory responses and restricts cell migration. This restricts the invasion of adjacent cellular tissues, prevents damage to bone and cartilage, and inhibits the tissue-destructive capacity of RA-FLS, ultimately reducing disease activity. Li et al. (77) demonstrated that H_2_S can inhibit HDAC6 gene expression and t HDAC6-induced inflammatory gene expression in RA-FLS. The H_2_S donor SPRC inhibited RA progression in a rat AIA model. SPRC achieved this by increasing cystathionine-γ-lyase (CSE) expression, decreasing synovial HDAC6 expression, and blocking the NF-κB signaling pathway. It is noteworthy that the studies on individual enzymes exhibit distinct differences in evidence hierarchy: the roles of PRMT1 and HDAC6 are supported by functional validation through gene knockout and selective inhibitors, indicating higher evidence strength; whereas research on CBP/p300 primarily relies on non-specific inhibitors or overexpression experiments, and their RA-specific function requires validation with more precise genetic tools. Furthermore, most studies have focused solely on the role of individual enzymes within a single cell type. The synergistic or antagonistic relationships among different enzymes in FLS, macrophages, and T cells remain to be elucidated.

The above discussion focuses on the function of individual modification enzymes; however, abnormalities in these enzymatic activities ultimately reshape higher-order chromatin structures. In recent years, the roles of chromatin accessibility, super-enhancers, and three-dimensional conformation in regulating inflammatory gene expression in RA have gradually been elucidated. Dysfunction in individual histone-modifying enzymes ultimately converges into systemic alterations in chromatin accessibility and three-dimensional conformation, forming the structural basis for RA synovial cells acquiring “inflammatory memory.” At the chromatin accessibility level, single-cell ATAC-seq studies further reveal transcriptional regulatory heterogeneity in RA FLS. Studies have demonstrated that in RA patients (78, 79), the chromatin accessibility of pro-inflammatory gene regulatory regions in FLS significantly increases and positively correlates with H3K27ac enrichment. This “chromatin relaxation” exhibits cell-subpopulation specificity—open chromatin regions in activated FLS subpopulations are enriched with binding motifs for NF-κB, STAT, and AP-1 family transcription factors, whereas resting FLS are characterized by accessibility to homobox transcription factors (e.g., PRRX1, ZFHX3). This differentiated chromatin state facilitates easier binding of transcription factors like NF-κB and STAT3 to target DNA sequences, establishing a cell-type-specific environment for sustained transcriptional permission. The Loh team (79) discovered that TNF stimulation induces a unique epigenetic memory in FLS: 280 “escape-inhibited genes” maintain sustained high expression, whereas the same genes in macrophages are suppressed after transient activation. This difference stems from FLS acquiring a persistent open chromatin state—H3K27ac enrichment and chromatin accessibility at FSG regulatory elements persist after stimulus withdrawal, providing a structural foundation for sustained gene expression. Notably, these regions enrich for NF-κB, IRF, and AP-1 binding motifs, suggesting that the chronic inflammatory phenotype of FLS arises from chromatin remodeling synergistically driven by multiple transcription factors. This discovery reveals an epigenetic mechanism underpinning RA chronicity: FLS convert transient inflammatory signals into persistent chromatin memory, creating an “inflammatory pedal effect.” Therapeutically, BET inhibitors can effectively “erase” this pathological chromatin memory by blocking recognition of acetylation modifications, suggesting that targeting epigenetic readers may represent a novel strategy to reset the inflammatory state of FLS and achieve disease modification. Chromatin accessibility heterogeneity exists not only between RA and healthy controls but also among FLS derived from different joints. The Hammaker team (80) discovered that FLS derived from hip and knee joints exhibit differences in chromatin accessibility even under baseline conditions, with these distinct regions enriched near the IL-6, IL-6R, and JAK1 genes. Notably, following IL-6 stimulation, STAT3 phosphorylation levels in knee FLS were significantly higher than in hip FLS, and this heightened responsiveness showed strong correlation with JAK1 protein levels. This suggests that joint-specific chromatin states may influence FLS cytokine responsiveness by regulating JAK-STAT pathway accessibility. It should be noted that the dynamic evolution of FLS subpopulations during disease progression and treatment response, along with the direct association between these chromatin state differences and clinical phenotypes (e.g., joint destruction severity, response to targeted therapies), warrant further investigation. The discovery of “escape suppressor genes” is primarily based on *in vitro* TNF stimulation models. Their actual presence in RA patients and their causal relationship with disease chronicity require validation through *in vivo* lineage tracing and intervention studies.

At the super-enhancer level, using osteoclast differentiation as an example, RANKL stimulation induces the establishment of 200 super-enhancers in CD14+ monocytes. These super-enhancers are enriched near genes such as NFATC1 and MYC, key regulators of osteoclasts, and are closely associated with gene sets related to bone diseases like RA. Conversely, SE near negative regulators (e.g., IRF8, KLF2) exhibit reduced H3K27ac enrichment upon RANKL activation (81). This paradigm demonstrates that dynamic SE remodeling is a key mechanism for cellular responses to microenvironmental signals and the acquisition of pathological phenotypes. A similar mechanism is emerging in RA FLS. Genome-wide epigenomic profiling of RA synovial tissue has revealed disease-specific regions of epigenetic regulation. The Ai team (82) conducted an integrated analysis of synovial tissues from RA and osteoarthritis (OA) patients, identifying over 30,000 epigenomically distinct markers. Among these, 9,804 H3K27ac differential sites and 9,813 open chromatin differential regions were detected. Notably, these differential regions co-localized with RA genetic risk loci (such as the LBH and FADS loci), suggesting that disease-specific enhancer activity abnormalities possess a genetic basis. Further studies (83) confirm the presence of multiple disease-associated super-enhancers in FLS, characterized by high levels of bromodomain protein 4 (BRD4) recruitment. These super-enhancers drive robust expression of core pro-inflammatory genes such as IL-6 and IL-1β, exhibiting high sensitivity to BRD4 inhibitors like JQ1. Notably, super-enhancer formation exhibits cell-type specificity, suggesting that targeting FLS-specific SEs may represent a novel strategy for precision epigenetic therapy. However, SE research in RA remains in its infancy. Currently identified SEs primarily originate from *in vitro* cultured FLS, and their actual presence and spatial distribution within *in vivo* synovial tissue remain unclear. The colocalization of SEs with RA genetic risk loci suggests causal potential, but functional validation remains limited—whether the therapeutic effects of BET inhibitors like JQ1 are indeed achieved by targeting specific SEs requires verification through more precise SE editing technologies. Furthermore, the distribution of SEs across different immune cell subsets (e.g., macrophages, T cells) and their roles in RA pathogenesis remain unexplored.

**Figure 5:**
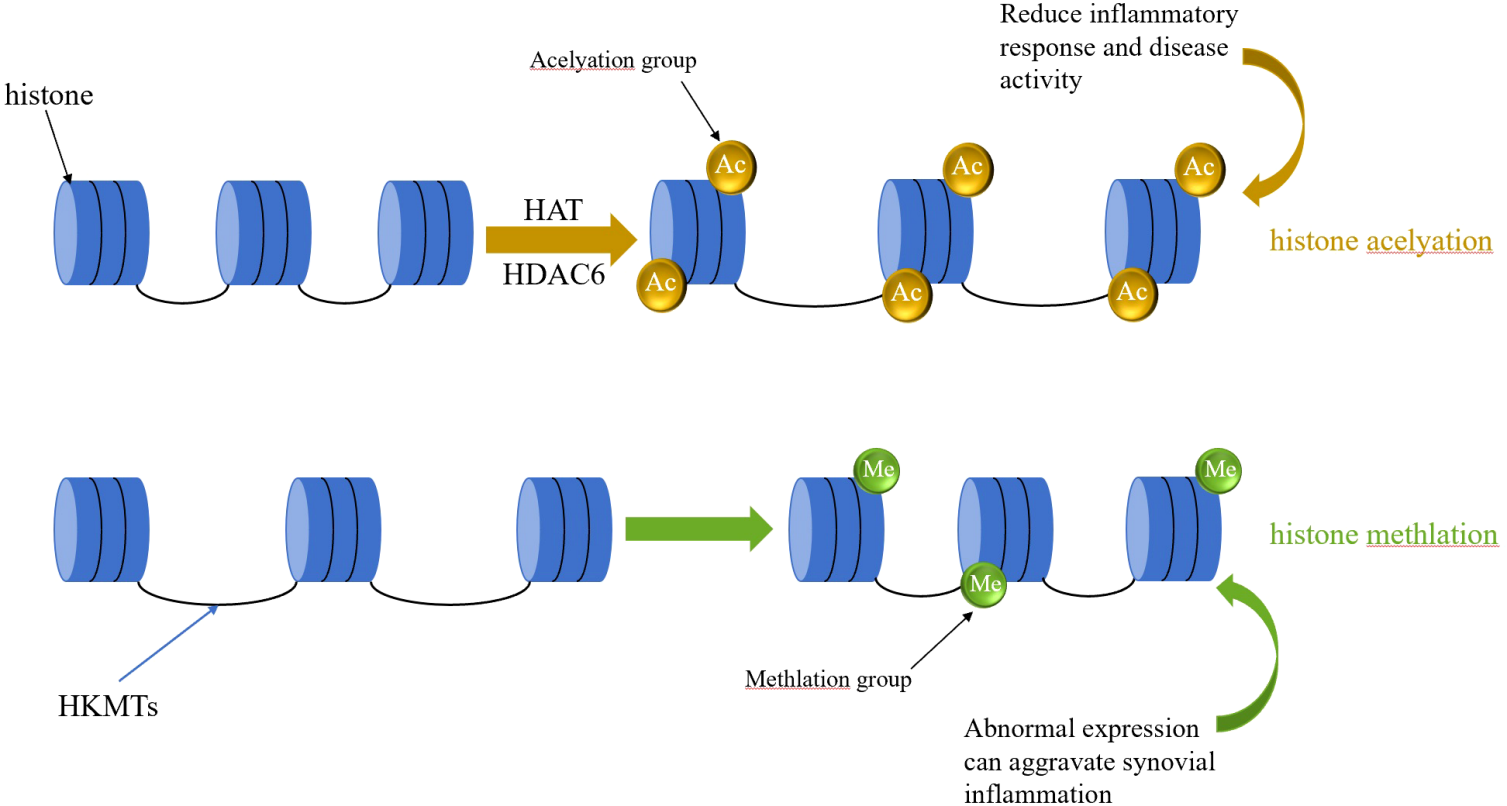
Schematic diagram of the pathogenic mechanism of histone modifications.

At the three-dimensional genomic level, Hi-C and derivative technologies (such as CHiC) reveal conformational rearrangements of inflammatory gene clusters in FLS. Ge et al. (84) found that TNF stimulation induces chromatin conformational reprogramming in FLS: approximately 800 chromatin interaction regions exhibit altered interaction strengths, positively correlated with expression changes in neighboring genes. Notably, interaction strength increased regardless of gene up- or downregulation, suggesting that chromatin remodeling does not simply “turn on” or “turn off” genes. Instead, it establishes new “accessibility platforms” for transcriptional regulation by reconfiguring enhancer-promoter interaction networks. Integrated analysis revealed that enhanced interaction regions were enriched for AP-1 binding sites, consistent with this transcription factor’s central role in inflammatory activation. Conversely, regions associated with downregulated genes were enriched for binding sites of BACH2 and homeobox/forkhead box family transcription factors, suggesting that developmental transcription factors may act as “inhibitory brakes” on FLS inflammatory responses. These findings reveal that 3D genomic remodeling constitutes a crucial structural foundation for FLS acquisition of a persistent inflammatory phenotype, while also providing direct evidence for cross-level regulation involving DNA methylation-chromatin conformation-transcriptional dysregulation. Three-dimensional genomic studies offer novel perspectives for understanding RA chronicity, yet face significant challenges. Current Hi-C/CHiC data primarily originate from *in vitro* cultured FLS populations, obscuring cellular heterogeneity; single-cell-resolution 3D genome technologies remain immature. While correlations between chromatin conformation alterations and gene expression changes have been demonstrated, causal relationships require direct validation through CRISPR-mediated loop editing. Furthermore, whether 3D genomic alterations can serve as diagnostic or prognostic biomarkers for RA, and whether drugs targeting chromatin conformation can be developed, remain frontier areas for future exploration. See **Figure 5** for details.

## Metabolic stress layer: hypoxia-driven metabolic–epigenetic coupling and vicious cycles in RA

Physiologically, articular cartilage tissue is in a hypoxic state due to its poor blood supply and the relatively enclosed nature of the joint cavity (85). The abnormal proliferation of FLSs relies on the hypoxic environment and the stimulation of growth factors in the synovial microenvironment (86). HIF-1α, an essential regulator in the HIF system for cellular responses to hypoxia, binds to hypoxia response elements (HREs) in DNA, thereby regulating the expression of multiple target genes and promoting the release of pro-inflammatory factors that intensify RA inflammation (87). In hypoxic conditions, the dysregulated generation of reactive oxygen species (ROS) in the human body activates RNA methylation sites, inducing a series of downstream inflammatory responses (88). Lin et al. (89) revealed that HIF-1α and its downstream effector molecule, Bcl-2/adenovirus E1B19kDa-interacting protein 3 (BNIP3), can trigger mitophagy. Deng’s team (90) put forward the idea that the relationship between autophagy and apoptosis depends on cell type and environment. OA-FLSs go through significant apoptosis under hypoxia. In contrast, FLSs experience extensive proliferation under the same conditions. Mitophagy is essential maintaining redox balance under hypoxia, which prevents a large-scale cell death. Consequently, FLSs can endure hypoxia, grow abnormally, and initiate a chain of inflammatory events. It should be noted that the aforementioned studies on the apoptotic differences between FLS and OA-FLS are primarily based on *in vitro* cell line models and lack *in situ* evidence at the human tissue level. Furthermore, the specific contribution of mitophagy to hypoxia tolerance in FLS has not been quantified, and its direct causal relationship with proliferative signaling requires further validation through methods such as gene knockout or pharmacological inhibition. Importantly, this layer is not limited to hypoxia signaling alone; rather, it focuses on how hypoxia-induced metabolic rewiring alters cofactor availability and oxygen-sensitive epigenetic enzyme activity, thereby stabilizing inflammatory transcriptional programs in RA.

**Figure 6:**
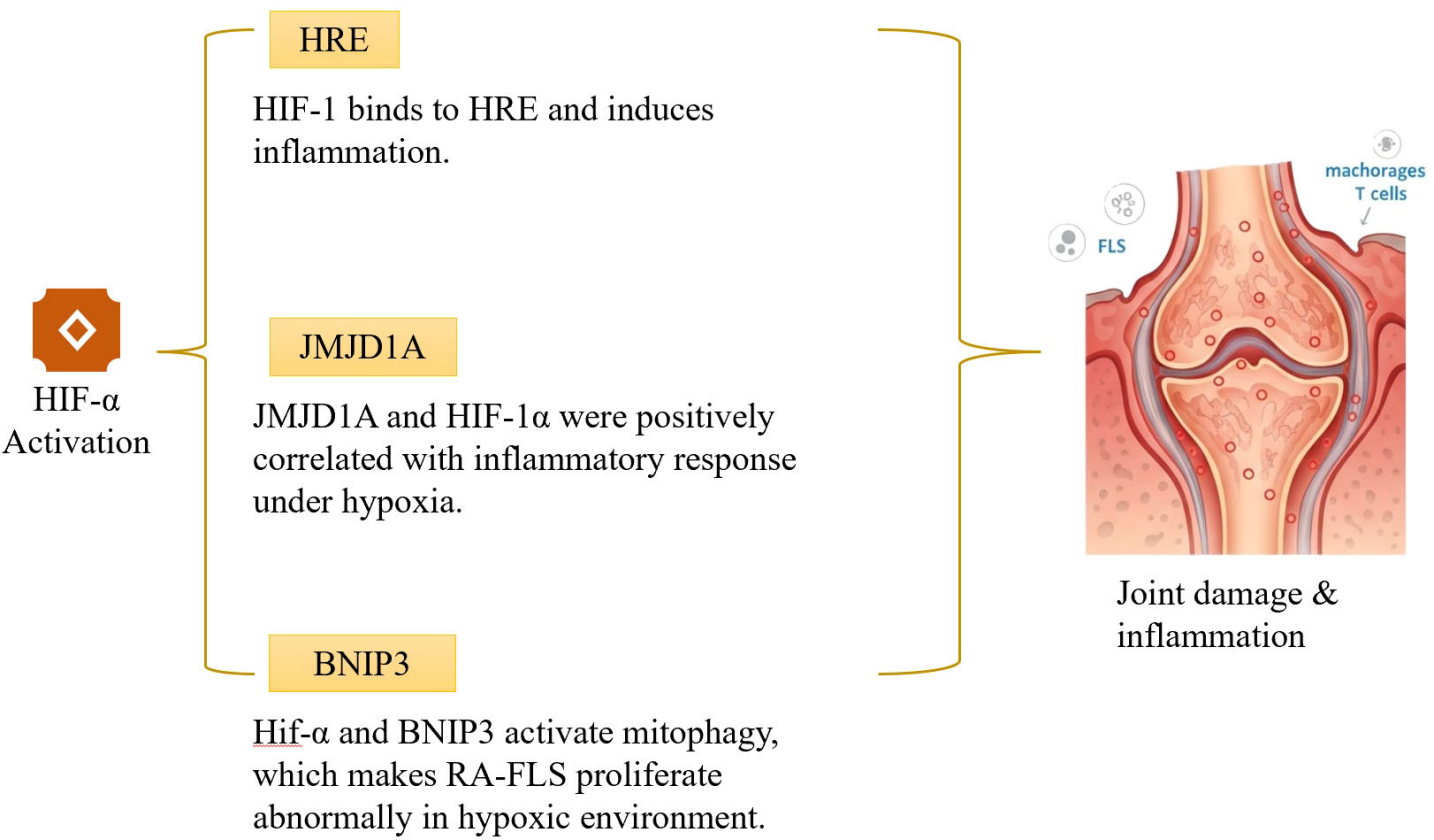
Pathogenic mechanism diagram of hypoxic microenvironment.

In addition to HIF-1α, the hypoxic microenvironment also directly regulates a superfamily of dioxygenases that share oxygen, α-ketoglutarate (α-KG), and Fe²^+^ as common substrates. This includes the TET family of DNA demethylases and the JmjC family of histone demethylases (91). These enzymes catalyze changes in epigenetic modifications through hydroxylation reactions, and their activity is strictly dependent on intracellular oxygen concentration (92). Under normoxic conditions, TET enzymes oxidize 5-methylcytosine (5mC) to 5-hydroxymethylcytosine (5hmC), initiating active DNA demethylation. In hypoxic states, oxygen becomes the rate-limiting substrate, causing a significant decline in TET activity: intracellular 5hmC levels decrease, while promoter methylation levels abnormally increase; and the removal of histone methylation marks is impeded, leading to chromatin state imbalance (93, 94). It is noteworthy that research on TET enzymes in RA remains in its infancy, with existing evidence primarily derived from oncology and developmental biology. The distribution patterns of 5hmC in RA synovial tissue, the specific expression of TET enzyme subtypes in FLS and immune cells, and their functional heterogeneity all await systematic elucidation. The Jmjc family of histone demethylases are not only directly inhibited by hypoxia but also forms a transcriptional co-regulatory network with HIF-1α. KDM6B (JMJD3), as a histone H3K27me3 demethylase, can be recruited by HIF-1α to hypoxia response element binding sites under intermittent hypoxia conditions. It regulates HIF-1 activity by demethylating H3K27me3, thereby activating the NF-κB pathway (95, 96). JMJD1C regulates the expression of key glycolytic enzymes hexokinase II (HK2), phosphoglycerate kinase 1 (PGK1), and lactate dehydrogenase A (LDHA). Under hypoxic conditions, JMJD1C expression is upregulated, leading to altered enzyme activity and establishing a feedback loop: hypoxia → altered JMJD1C activity → metabolic adaptation. (PGK1), and lactate dehydrogenase A (LDHA). Under hypoxic conditions, JMJD1C expression increases, altering enzyme activity and forming a positive feedback loop: hypoxia → altered JMJD1C activity → metabolic adaptation → epigenetic remodeling → sustained inflammation. This may promote downstream epigenetic remodeling and sustained inflammation. In a hypoxic model of pulmonary arterial hypertension, JMJD1C knockdown significantly reduced lactate accumulation and inhibited STAT3 signaling activation. Conversely, STAT3 overexpression reversed the effects of JMJD1C deficiency, confirming that JMJD1C drives glycolysis and cell proliferation by activating STAT3 signaling (97). Although this evidence originates from cardiovascular diseases, the hypoxia-driven metabolic-epigenetic regulatory mechanism is highly conserved, suggesting that the JMJD1C-glycolysis axis may similarly participate in inflammatory amplification and disease chronicity in FLS. It is important to emphasize that this inference requires direct functional validation in RA-specific cells, including the expression levels of JMJD1C in FLS, phenotypic changes following JMJD1C knockdown, and the causal relationship with STAT3 signaling. Furthermore, Jumonji domain-containing protein 1A (JMJD1A), a histone demethylase, serves as a key epigenetic marker. Under hypoxia, the expression levels of both JMJD1A and HIF-1α increase and show a positive correlation with inflammatory responses (98). See **Figure 6** for details.

## Environmental interaction layer: microbiota-host epigenetic dialogue regulates RA immune homeostasis

Microorganisms—encompassing bacteria, fungi, viruses, and other taxa—include both beneficial and pathogenic species. Current research implicates gut dysbiosis and reduced microbial diversity in RA initiation (99). The gut microbiota influences host immune homeostasis through its metabolites, and its regulatory mechanisms can be understood through across two progressive levels.

At the level of immunometabolic effects, short-chain fatty acids (SCFAs) such as butyrate and propionate exert immunomodulatory actions by activating G protein-coupled receptors (GPR41/43). For instance, butyrate activates GPR43 on T cell surfaces, promoting IL-10 production and Treg differentiation (100). This pathway does not involve direct inhibition of epigenetic enzymes but instead indirectly influences immune cell function through receptor-mediated signal transduction. Although such studies are abundant in RA, they essentially represent immunometabolic regulation rather than direct epigenetic mechanisms. Moreover, most evidence derives from *in vitro* cell experiments or animal models, and the actual effects in RA patients require further validation.

At the level of direct epigenetic enzyme inhibition, butyrate acts as a broad-spectrum HDAC inhibitor. In Treg cells, butyrate treatment of naive T cells enhances histone H3 acetylation levels in the Foxp3 locus promoter region and conserved non-coding sequences. Additionally, propionate has been demonstrated to inhibit HDAC activity, thereby reducing the production of pro-inflammatory cytokines (TNF-α, IL-6) (101). This evidence establishes a direct causal chain: gut microbiota metabolites → HDAC inhibition → altered histone acetylation → immune regulation. However, it is worth noting that the HDAC family comprises 11 members, and the selective inhibition of different subtypes by butyrate, along with its specific targets in RA, still requires systematic elucidation.

**Figure 7:**
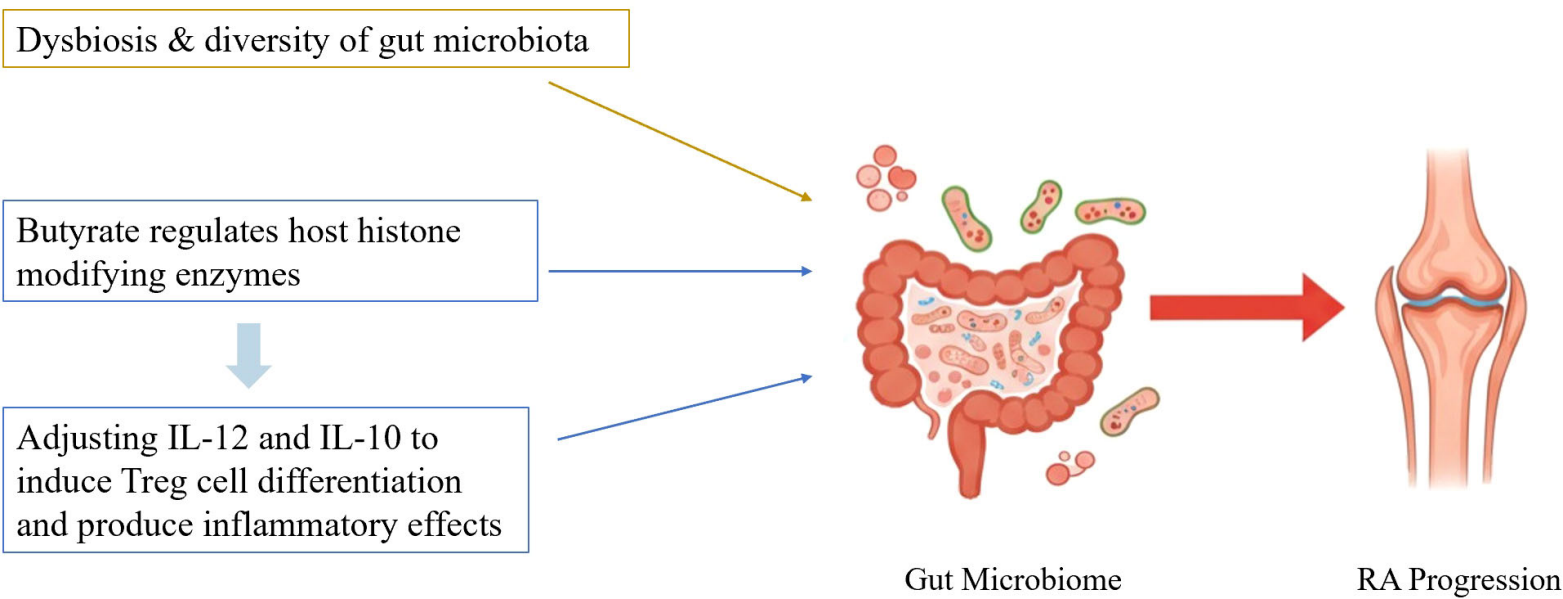
Pathogenic mechanisms of gut microorganisms.

Research directly validating the microbiota-epigenetic causal mechanism in the RA field remains extremely limited. Based on conserved mechanisms observed in other disease models: In esophageal cancer, Fusobacterium nucleatum exhibits a significant association with LINE-1 hypomethylation, thereby influencing host DNA methylation and altering disease prognosis (102); In obese models, butyrate modulates inflammatory pathways downstream of MAPK and NF-κB by inhibiting HDAC9 and HDAC2 (103). These findings suggest that similar metabolite-epigenzyme interactions may exist in RA, but this possibility requires functional validation in RA-specific cells (e.g., FLSs and autoreactive T cells). However, these discoveries originate from non-RA models and predominantly represent correlational studies, lacking direct evidence of causality. For instance, whether the HDAC inhibitory effects observed in obesity models apply to FLS or autoreactive T cells requires functional validation. Additionally, pathogenic bacteria modulate host epigenetics to survive, replicate, and evade immune destruction following infection. This impairs the immune system’s ability to eliminate pathogens (104), suggesting that dysregulated cross-species epigenetic transmission may serve as a key molecular bridge linking environmental factors to RA autoimmunity. See **Figure 7** for details.

## Evidence synthesis and strength grading

To systematically evaluate the evidence strength of evidence in existing RA epigenetics research, we conducted a comprehensive analysis of key molecules discussed throughout the literature (**Table 5**). Evidence grading was based on the follows criteria:

**Table 5:** Summary of evidence strength grading.

Mechanism	Key molecule/modification	Sample source	Evidence type	Evidence strength	Remarks
miRNA	miR-155↑	RA patient T cells, FLS cell lines, CIA mice	Correlation analysis + functional validation (knock down)	★★★★☆	Multi-model validation, strong evidence
miRNA	miR-146a↑	RA patient FLS cell lines, CIA mice	Correlation analysis + functional validation (*in vitro*)	★★★★☆	Multi-model validation, strong evidence
m^6^A	METTL3↑	FLS cell line, CIA mice	Functional validation (knock out)	★★★★☆	Opposite effect in macrophages requires further investigation
DNA methylation	High methylation of FOXP3 promoter	RA Treg cells	Correlation analysis + functional validation (inhibitor)	★★★★☆	Partial causal validation
Histone Modification	HDAC3 Activity↑	FLS cell line、AIA mice	Functional Validation (Inhibitor)	★★★★☆	Microbiome metabolite butyrate can inhibit
Hypoxia	JMJD1C↑	Hypoxia model of pulmonary hypertension	Correlation analysis + functional validation (knock out)	★★★☆☆	Mechanism partially derived from other disease models
Microbiota	Butyrate↓	Treg cells in RA patients	Correlation + functional validation (*in vitro*)	★★★☆☆	Mechanism partially derived from other disease models

★★★★★: Multi-model validation with robust functional and causal evidence
★★★★☆: Functional validation supported, but lacking human primary cell or single-cell data
★★★☆☆: Primarily correlational evidence with partial functional indications
★★☆☆☆: Correlation only, lacking functional validation
★☆☆☆☆: Preliminary findings requiring further validation

Overall, RA epigenetics research exhibits a pronounced imbalance in evidence hierarchy: mechanisms involving miR-155, METTL3, HDAC3, and others have undergone multi-model functional validation with high evidence strength. Direct evidence for microbiota-epigenetic interactions is particularly weak, with most conclusions derived from other disease models or *in vitro* experiments, urgently requiring functional validation in RA-specific cells. Future research should integrate CRISPR epigenomic editing, organoid models, and prospective cohorts to elevate associative findings into causal mechanisms.

## Summary and outlook

The pathogenesis of RA remains incompletely elucidated, with epigenetic regulation emerging as a pivotal contributing factor. This review summarizes key epigenetic modulators implicated in RA pathogenesis, with a focused analysis of miRNAs, RNA methylation, DNA methylation, gut microbiota, and hypoxia. As crucial regulators of post-transcriptional gene expression, miRNAs exert their functions by targeting and repressing mRNAs encoding pro-inflammatory mediators, thereby governing inflammatory responses and synovial hyperplasia—two hallmark features of RA (105). RNA methylation involves the deposition of methyl groups onto RNA molecules to reshape gene expression outcomes; m^6^A methylation, in particular, modulates the post-transcriptional fate of its RNA targets (106), and thus plays a vital role in directing immune cell differentiation and promoting synovial invasion in RA. DNA methylation modifies chromatin structure through methyl group addition at CpG sites, leading to context-dependent gene silencing or activation and subsequent inflammatory activation (107). Among these, LRPAP1 stands out as the gene most tightly linked to RA pathogenesis and has been validated as a novel therapeutic target for the disease. In the realm of histone modifications, HATs and HDACs have been the most extensively characterized; these enzymes dynamically add or remove acetyl groups from histone tails to fine-tune gene transcription (108). Hypoxia signaling also contributes to RA pathogenesis: HIF-1α binds to genomic HREs to unleash a pro-inflammatory factor storm that amplifies inflammatory cell infiltration. Additionally, hypoxic microenvironments activate canonical hypoxia regulatory factors to induce mitochondrial autophagy, a process that drives aberrant synovial cell proliferation and kickstarts pathogenic inflammatory cascades in RA. For details, see the evidence summary table (**Table 6**).

**Table 6:** Regulatory direction and evidence strength of key epigenetic molecules in RA.

Molecule/modification	Cell type/model	Experimental approach	Molecular mechanism	Effect on RA	Evidence type	Contradictions/remarks
miR-155	RA patients T cells	Overexpression	Targets SOCS1→STAT3 activation	Promotes disease	Association + functional validation (knock down experiments)	Consistent
miR-146a	RA patient T cells (early stage)	Overexpression	Targets IRAK1/TRAF6 → NF-κB inhibition	Suppresses disease	Association + functional validation	Downregulated in late-stage FLS, losing protective effect
METTL3	RA macrophage	overexpression	m^6^A modification IRAK-M→alternative NF-κB activation	disease suppression	functional validation (knockout)	potentially pro-inflammatory in FLS (Further research needed)
FTO	FLS	overexpression	m^6^A modification → reduced ADAMTS15	Promotes disease	functional validation	May act differently in T cells
FOXP3 promoter methylation	RA Treg cells	High methylation	Transcriptional silencing → Treg dysfunction	Promotes disease	Association + functional validation (5-aza treatment)	Consistent
PRMT1	FLS	overexpression	Histone arginine methylation → Proliferation/anti-apoptosis	Promotes disease	Functional validation (knock out)	(Originally placed in DNA methylation section, now removed)
MLL1	RA-SF	overexpression	Elevated H3K4me3 → Activation of IL-6, etc.	Promotes disease	Functional validation (knock out)	Consistent
HIF-1α	FLS(hypoxia)	Stable expression	Activates Bcl-2/BNIP3 → Initiates mitochondrial autophagy	Promotes disease	Functional validation	Forms hypoxia-epigenetic vicious cycle
JMJD1C	Hypoxia model (pulmonary hypertension)	Upregulation	Activates glycolytic enzymes → STAT3 signaling	Promotes disease	Functional validation (knock down)	Requires further validation in RA
Butyrate	Treg cells	Treatment	Inhibition of HDAC3 → Foxp3 H3K9ac↑	Disease suppression	Functional validation	Still requires validation in RA

The epigenetic regulatory network in RA exhibits multidimensional cross-interaction mechanisms.

DNA methylation drives miRNA dysregulation: In RA model rats, upregulation of DNMT1 expression leads to hypermethylation of the CpG island in the miR-152 promoter region, resulting in significant downregulation of miR-152 expression. Notably, miR-152 directly targets the 3’ UTR of DNMT1 to inhibit its expression, forming a double negative feedback loop: DNMT1 hypermethylation silences miR-152 → reduced miR-152 releases inhibition on DNMT1 → further elevation of DNMT1. This circuit promotes FLS proliferation and inflammatory cytokine secretion by activating the Wnt signaling pathway (targeting SFRP1/SFRP4), converting environmental stimuli (such as smoking) into persistent epigenetic memory (109). Microbiome metabolites regulate HIF-1α through dual mechanisms: Short-chain fatty acids (e.g., butyrate) not only inhibit HDAC activity but also activate the STAT3/HIF-1α signaling pathway via GPR41/43 receptors, thereby modulating macrophage function (110). In hypoxic microenvironments, HIF-1α serves as a metabolic-epigenetic regulatory hub. On one hand, it recruits p300/CBP to enhance H3K27ac modifications in pro-inflammatory gene enhancer regions (111). On the other hand, it regulates the expression of key glycolytic enzymes, forming a positive feedback loop: “hypoxia → metabolic reprogramming → epigenetic remodeling → sustained inflammation.” Together, these mechanisms constitute a vicious cycle of “microbiome metabolism → hypoxia response → epigenetic remodeling.”

Given the pivotal role of gut microbiota in the epigenetic regulation of RA, the holistic principles and preventive medicine philosophy inherent to traditional Chinese medicine (TCM) provide a distinctive framework for targeting microbiota-host epigenetic interactions. Berberine—also referred to as berberine hydrochloride in some pharmacological formulations (rather than coptisine)—exhibits substantial gut accumulation; acting as a gut microbiota modulator, it enhances the production of the microbial metabolite butyrate and can even regulate physiological hypoxia in collagen-induced arthritis (CIA) rats via HIF-1α upregulation (112). Similarly, Scutellaria baicalensis-based preparations (formulated for heat-clearing and arthralgia-relieving effects) modulate gut microbiota composition in CIA rats, reducing lipopolysaccharide (LPS) biosynthesis and its translocation into peripheral circulation, thereby dampening systemic inflammatory responses (37). Notably, TCM interventions can target gut microbiota ecology and host epigenetic modifications through bioactive components derived from single medicinal herbs. In contrast, TCM formulae exert multi-target synergistic effects to reshape gut microbiota homeostasis and rectify epigenetic dysregulation, which may yield innovative intervention strategies targeting RA-associated epigenetic regulatory networks. Recent research (113), building upon the theory that co-inhibitory receptors regulate T cell immunity, proposes that the traditional Chinese medicine (TCM) approach of “tonifying the body’s inherent resistance” may exert therapeutic effects by modulating T cell immune balance. This offers a novel theoretical perspective for the integrated treatment of rheumatoid arthritis (RA) combining Chinese and Western medicine. This concept potentially intersects with the epigenetic regulatory networks discussed in this paper—epigenetic reprogramming of T cell function may represent one of the molecular foundations underlying the “tonifying the body’s inherent resistance” approach. Furthermore, mechanism studies on traditional therapies like acupuncture provide fresh evidence for epigenetic regulation. Recent studies (114) reveal that acupuncture induces characteristic alterations in gene co-expression networks in osteoarthritis patients, suggesting it may exert therapeutic effects by regulating specific gene networks. These findings open new avenues for exploring the epigenetic mechanisms of TCM external therapies—whether acupuncture influences FLS and immune cell functions by modulating DNA methylation, histone modifications, or non-coding RNAs represents a promising interdisciplinary research frontier. Collectively, these TCM-based approaches hold considerable translational potential and research merit, while also offering a promising avenue to address current bottlenecks in RA therapeutic development.

Although epigenetic interventions show substantial preclinical promise in RA, RA-specific clinical validation remains limited for most candidate strategies, including DNMT inhibitors, HDAC-directed agents, and RNA methylation modulators. A major challenge is that many epigenetic regulators are broadly expressed across tissues and cell types; therefore, systemic administration may produce off-target epigenomic effects, narrow therapeutic windows, and unpredictable long-term safety profiles. In addition, delivery specificity remains a central bottleneck, particularly when the therapeutic goal is to reprogram pathogenic synovial fibroblasts, macrophage subsets, or autoreactive lymphocytes without disrupting homeostatic immune function. Future translational progress will likely depend on integrating cell- and tissue-targeted delivery systems with biomarker-guided patient stratification, including molecular endotypes, synovial phenotypes, and single-cell/multi-omics-defined pathogenic subpopulations. In this context, the most clinically feasible near-term strategy may not be broad epigenetic reprogramming, but precision modulation of selected epigenetic nodes in biologically defined patient subsets.

To address these barriers, future research should prioritize the following directions:

1. Development of high-resolution epigenetic atlases: Deploy integrated single-cell multi-omics and spatial omics technologies to decode characteristic epigenetic modifications across different pathological stages of RA. This includes constructing dynamic epigenetic profiles in distinct tissue microenvironments (e.g., synovium and intestinal mucosa), defining the spatial distribution and regulatory signatures of key pathogenic cell populations, and identifying clinically actionable epigenetic targets linked to disease progression and treatment response.
2. Tissue-/cell-targeted epigenetic intervention strategies: Develop delivery systems with improved tissue and cell specificity (e.g., liposomes, polymeric nanoparticles, or ligand-directed carriers) to reduce off-target effects and increase local efficacy. Beyond conventional delivery platforms, synergistic strategies combining microbiota editing with epigenetic modulation may offer additional translational value, including gut-targeted systems that locally deliver or induce epigenetic regulators (e.g., butyrate and related metabolites) to shape systemic immune responses.

More broadly, translating epigenetic insights into clinical benefit will require rigorous causal validation, standardized methodological frameworks, and improved biosafety evaluation for targeted delivery systems. At present, clinically available RA therapies primarily suppress inflammation and slow tissue damage rather than reverse core pathogenic programs. The long-term goal of epigenetic therapy is therefore to move from broad “global modification” toward precision, context-aware intervention. With continued advances in single-cell epigenomics, spatial biology, biomarker-guided stratification, and targeted delivery technologies, epigenetic strategies may ultimately contribute to disease-modifying and personalized RA treatment.

Although epigenetic interventions show substantial preclinical promise in RA, RA-specific clinical validation remains limited for most candidate strategies, including DNMT inhibitors, HDAC-directed agents, and RNA methylation modulators. A major challenge is that many epigenetic regulators are broadly expressed across tissues and cell types; therefore, systemic administration may produce off-target epigenomic effects, narrow therapeutic windows, and unpredictable long-term safety profiles. In addition, delivery specificity remains a central bottleneck, particularly when the therapeutic goal is to reprogram pathogenic synovial fibroblasts, macrophage subsets, or autoreactive lymphocytes without disrupting homeostatic immune function. Future translational progress will likely depend on integrating cell- and tissue-targeted delivery systems with biomarker-guided patient stratification, including molecular endotypes, synovial phenotypes, and single-cell/multi-omics-defined pathogenic subpopulations. In this context, the most clinically feasible near-term strategy may not be broad epigenetic reprogramming, but precision modulation of selected epigenetic nodes in biologically defined patient subsets.
